# Tandem duplication-driven expansion and UV-B stress adaptation of the *LHC* gene family in *Artemisia annua* L.

**DOI:** 10.1186/s12870-026-09370-4

**Published:** 2026-06-29

**Authors:** Xiaoxia Ding, Danchun Zhang, Siyu Zhao, Shengye Bao, Rongrong Zou, Jieting Chen, Lu Gong, He Su, Qiong Luo, Hengyu Pan, Lizhi Wang, Shilin Chen, Zhihai Huang, Baosheng Liao

**Affiliations:** 1 School of Chinese Materia Medica, Tianjin University of Traditional Chinese Medicine, Tianjin, 300193 China; 2 The Second Clinical College of Guangzhou University of Chinese Medicine, Guangzhou, 510006 China; 3 School of Traditional Chinese Medicine, Guangdong Pharmaceutical University, Guangzhou, 510006 China; 4 Yunmeng People’ s Hospital, Xiaogan, Hubei 432500 China; 5 Institute of Herbgenomics, Chengdu University of Traditional Chinese Medicine, Chengdu, 611137 China

**Keywords:** *Artemisia annua*, *LHC*, *ELIP*, UV-B, Dynamic evolution

## Abstract

**Background:**

*Artemisia annua* L., is the primary natural source of the antimalarial drug artemisinin. In nature, fluctuating light is a major environmental stress that affects plant growth and artemisinin biosynthesis. Although the light-harvesting chlorophyll a/b-binding (*LHC*) superfamily plays a key role in mediating plant responses to fluctuating light, systematic research of this gene family in *A. annua* has not yet been conducted, limiting our understanding of light adaptation in this medicinally important species.

**Results:**

This study investigated the evolutionary dynamics and functional adaptation of the light-harvesting chlorophyll a/b-binding (*LHC*) superfamily in *A. annua*, with a focus on the early light‑induced protein (*ELIP*) subfamily. Comparative genomics of 24 plant species showed that the *LHC* superfamily recently expanded in the examined Asteraceae lineages through duplication events. In *A. annua*, 229 *LHC* genes identified from four haplotype genomes comprised 205 allelic and 24 haplotype-specific loci, with the *ELIP* subfamily expanding significantly via tandem duplication. Notably, compared to non-Asteraceae plants, *ELIPs* exhibited a uniform single-exon architecture, indicating it is a genomic feature unique to Asteraceae plants. Population genomics of 41 individuals showed dynamic copy number variations ranging from 1 to 4 copies per locus. Interestingly, a structurally disrupted *ELIP* allele remained transcriptionally active and produced long aberrant transcripts, showing that this subfamily is still actively evolving. Under UV-B stress, *AaELIP* loci showed synchronized induction trend but differed in expression levels, suggesting a division into major and auxiliary roles within the expanded tandem cluster. Overall, while the response of *ELIPs* to light stress is evolutionarily conserved, this dramatic expansion and structural streamlining of *AaELIPs* may represent a key evolutionary adaptation that enhances the plant’s ability to cope with intense light and radiation stress.

**Conclusions:**

Collectively, this study demonstrates a significant expansion of the *LHC* superfamily in *A. annua*, especially within the *ELIP* subfamily, as well as its robust response to UV-B treatment, underscoring the essential role of *ELIPs* in mediating light stress responses. These findings provide a valuable foundation for future research to uncover the molecular mechanisms underlying *A. annua*’s adaptation to complex light environments.

**Supplementary Information:**

The online version contains supplementary material available at https://doi.org/10.1186/s12870-026-09370-4.

## Background

*Artemisia annua* L. is a globally distributed traditional Chinese medicinal plant and the sole natural source of artemisinin, a frontline antimalarial drug [1–4]. As a prominent heliophyte, *A. annua* frequently encounters complex, dynamic, and intense light in its natural habitats. Extreme irradiance can induce a 14% reduction in photosynthetic capacity due to photoinhibition, alongside a substantial upregulation of non-photochemical dissipation (NPQ) [5]. Beyond serving as an energy source and developmental signal [6], fluctuating light acts as environmental stress that elevates reactive oxygen species (ROS) concentrations, which subsequently influences leaf artemisinin content [5, 7, 8]. Because maintaining metabolic productivity under these variable conditions depends on a robust genetic basis, understanding the molecular mechanisms underlying the plant’s response to these complex light environments is essential to clarify its ecological resilience and environmental adaptation of this medicinally important species.

Central to plant photoprotective and light acclimation are members of the light-harvesting chlorophyll a/b-binding (*LHC*) superfamily [9]. This superfamily is structurally and functionally divided into classic antenna proteins for light capture under normal conditions, and stress-induced LHC-like proteins dedicated to mitigating photodamage [9, 10]. Under environmental stress, most LHC-like proteins—encompassing one-helix proteins (OHPs), stress enhanced proteins (SEPs), LHC-like proteins (Lils), and early light-induced proteins (ELIPs)—are specifically upregulated to dissipate excess energy through mechanisms such as NPQ, thereby preventing the overproduction of ROS [9, 11, 12]. Among them, *ELIPs* were the first described to accumulate during early thylakoid development and light stress, subsequent photobiological research has demonstrated that their stress-induced accumulation closely correlates with the accelerated degradation and turnover of the Photosystem II (PSII) reaction center D1 protein during photoinhibition [5].

The genomic composition, duplication origins, and functional roles of the *LHC* superfamily have been systematically characterized in model plants and crops, including *Arabidopsis thaliana* [13], *Oryza sativa* [13], and *Manihot esculenta* [14]. Functional analyses in *Gossypium hirsutum* further demonstrated that specific members, such as *GhLhcb2.3*, influence chlorophyll a synthesis [15]. Across broader evolutionary scales from algae to angiosperms, comparative genomics has elucidated phylogenetic relationships, copy number variations (CNVs), and motif distributions within this gene family [16]. Beyond general light harvesting, the *ELIP* subfamily is known to participate in diverse environmental responses, such as high-light-induced accumulation in *Pisum sativum* [5], desiccation and salinity tolerance in *Tortula ruralis* [10], and cold-induced photoprotection in *Chlamydomonas reinhardtii* [12]. Although *ELIPs* have been implicated in abiotic stress tolerance across various species [5, 10, 12, 17, 18], a systematic analysis of this superfamily within the stress-resilient Asteraceae family, particularly within *A. annua*, has not yet been conducted.

This study systematically investigated the *LHC* superfamily across 24 plant species through comparative genomics, revealing a recent, duplication-driven expansion of the superfamily within *A. annua* and its close relatives. In *A. annua*, 229 *LHC* genes were characterized from four haplotype genomes. Importantly, the *ELIP* subfamily was found to have undergone extensive tandem duplication and evolved a conserved single-exon structure. Population genomics across 41 diverse individuals further revealed dynamic copy number variations within natural populations. Notably, a structurally disrupted *ELIP* allele remained transcriptionally active and produced long aberrant transcript isoforms, highlighting the active evolution of this subfamily. Transcriptomic profiling under UV-B stress demonstrated a strong, synchronized induction trend across *AaELIP* loci, though their absolute expression levels diverged, suggesting a potential division into major and auxiliary roles within the expanded tandem cluster. Collectively, these findings demonstrate that recent gene duplications, structural evolution and expression divergence have shaped a specialized, UV-B-responsive *ELIP* subfamily in *A. annua*, which may enhance photoprotection under high-light (HL) stress and provide a valuable genomic foundation for elucidating light‑stress adaptation mechanisms in this medicinal species.

## Materials and methods

### *LHC* genes identification and manual correction

Annotated proteins from four haplotype genomes (LQ9P0, LQ9P1, HAN1P0, and HAN1P1) of two *A. annua* strains—HAN1 (high-artemisinin production) and LQ-9 (low-artemisinin production)—were downloaded from the Global Pharmacopoeia Genome Database [19] (GPGD, http://www.gpgenome.com/). Guided by the *A. thaliana* LHC information (Table S1), candidate *A. annua* LHCs containing any of the three target domains (PF00504, PF00762, or PF13806) were initially identified. To ensure comprehensive identification and minimize potential omissions, a genome-wide BLASTP search [20] was subsequently conducted using all LHC proteins of *A. thaliana* as queries against the four haplotype protein annotation datasets of *A. annua*. All retrieved candidates were then validated using three databases (CDD, Pfam, and InterPro) [21–23]. For classification, most LHCs harbor the PF00504 domain, whereas FCII and Psb33 are strictly defined by their specific domains (PF00762 and PF13806, respectively) combined with a C-terminal chlorophyll-binding region. Candidate *LHC* genes were reviewed and manually corrected using the Apollo browser [24]. For comparative genomics analyses, *LHC* genes from 23 additional plants were identified and curated using the same pipeline (Table S2). Allelic relationships among *A. annua LHC* loci were determined based on an amino acid sequence identity threshold of ≥ 92%. The number of amino acids, isoelectric point (pI), and molecular weight (MW) were calculated using ProtParam (https://web.expasy.org/protparam/). The multiple sequence alignment and pairwise similarity calculation of full-length LHC proteins were performed using Clustal Omega v1.2.4 [25]. Phylogenetic trees were visualized and annotated using the R package ggtree v4.2.0 [26]. KaKs calculator 3.0 [27] was used to calculate Ks value.

### Characterization and copy number evaluation of *AaELIPs*

Protein sequences of AaELIPs were aligned against *A. thaliana* orthologs (ELIP1 and ELIP2) using Clustal Omega [25], and the resulting alignments were visualized in R. The genomic resequencing reads from 41 *A. annua* samples were used to evaluate the copy number of *ELIP* loci (Table S3). High-throughput sequencing data were aligned to four haplotype genomes with bowtie2 (version 2.4.4) [28]. Reads mapping to the *ELIP* genomic regions were extracted and re-aligned to the reference *ELIP* loci to refine mapping accuracy. Sequencing depth was calculated with bedtools (version 1.6) [29]. For each sample, the average sequencing depth on the whole genome was calculated and defined as global sequencing depth. The sequencing depth of each individual *ELIP* locus for each sample was normalized by dividing by the global sequencing depth. The normalized sequencing depth was defined as the relative sequencing depth, which represented copy number in this study.

### Validation of *ELIP* structural variations

Genomic DNA and total RNA were extracted from tissue‑culture seedlings of the strain LQ‑9 using a modified 3×CTAB protocol [30] and FastPure Plant Total RNA Isolation Kit (Vazyme), respectively. The concentration and purity of DNA and RNA were determined using the NanoDrop2000 ultra-micro ultraviolet spectrophotometer (Thermo Scientific, MIT, USA). Gene-specific primers were designed using Primer Premier 5 (Table S4). PCR amplifications were performed in 25 µL reactions containing 12.5 µL of 2× Phanta^®^ Flash Master Mix (Dye Plus), 1.0 µL each of forward and reverse primers (10 µM), 1.0 µL of genomic DNA, and nuclease-free water up to 25 µL. The PCR amplification conditions were listed in Table S4. Amplified products were separated on a 1.0% agarose gel and sequenced on the Oxford Nanopore Technologies (ONT) platform. Sequencing reads were mapped to the structurally disrupted *ELIP* locus within the LQ9P1 genome using Minimap2 (version 2.17) [31], and the gene structure was visualized and manually verified using the Integrative Genomics Viewer (IGV) [32].

### Plant materials and culture conditions

Seeds of *A. annua* were collected from the Institute of Chinese Materia Medica, China Academy of Chinese Medical Sciences, and authenticated by Professor Li Xiang. Following germination on moist filter paper in the dark at 20 ℃ and 60% relative humidity (RH), seedlings were transferred to plastic pots and grown under ambient conditions for four weeks, followed by three months of cultivation in a greenhouse at Guangzhou University of Chinese Medicine. Three uniform, healthy seedlings (designated as S1-S3) were acclimatized for one week in a controlled growth chamber (16/8 h light/dark photoperiod, 60% RH) prior to parallel treatment. On consecutive days, plants were subjected to either white light (WL) or ultraviolet-B (UV-B) treatment for 0, 2, 4, 6, or 8 h (designated as WL-0 to WL-8 and UV-0 to UV-8, respectively). Plants were rotated every 15 min during exposure to ensure uniform irradiation. At each designated time point (10:00, 12:00, 14:00, 16:00, and 18:00), fully expanded leaves were collected from three specific stem positions (3, 5, and 10 cm below the apex). The collected tissue samples were immediately frozen in liquid nitrogen, and stored at -80 ℃ for further analyses.

### RNA extraction, transcriptome sequencing and analysis

Total RNA was extracted from *A. annua* samples using the FastPure Plant Total RNA Isolation Kit (Vazyme). RNA quality was verified, and sequencing libraries were prepared and sequenced on an Illumina platform in 150 bp paired-end mode. Clean reads were mapped to LQ9P0 reference genome with HISAT2 (version 2.1.0) [33], and gene expression was calculated with StringTie (version 1.3.4d) [34]. Differentially expressed genes (DEGs) between treatments were identified using DESeq2 with |log_2_(FoldChange)| > 1 and an adjusted p-value < 0.05 [35]. Gene Ontology (GO) enrichment analysis of DEGs was performed using the clusterProfiler package [36]. For integrated expression analysis, publicly available RNA-seq datasets for *A. annua* under various treatments were downloaded from NCBI and integrated with our unpublished stress-response transcriptome datasets (Table S5).

## Results

### *LHC* superfamily expansion within the examined Asteraceae species driven by recent gene duplications

**Fig. 1:**
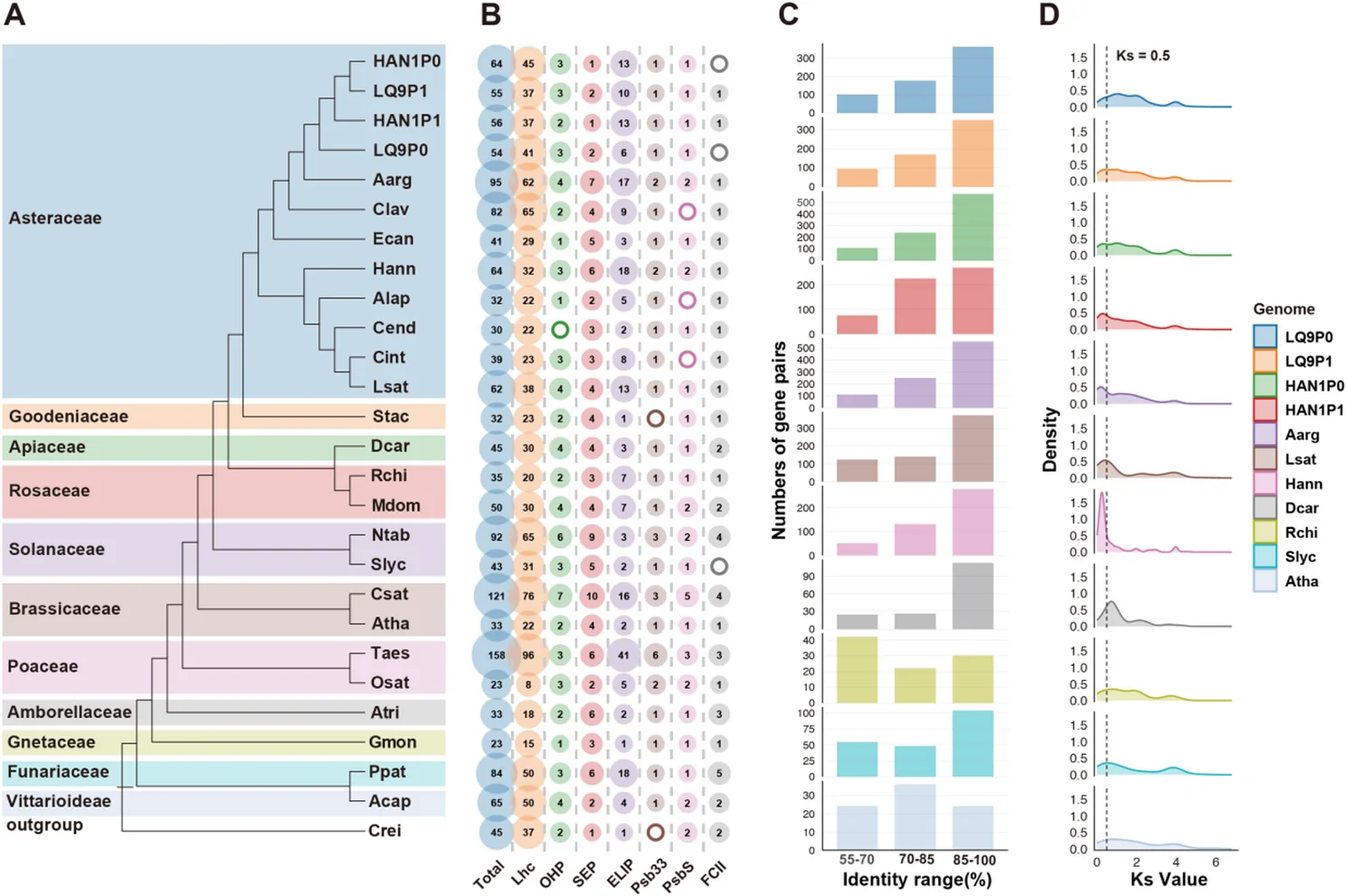
Comparative genomic and evolutionary analysis of *LHC* superfamily. **A** Phylogenetic tree of 24 species: HAN1P0/HAN1P1/LQ9P0/LQ9P1 *A. annua*, Aarg *A. argyi*, Clav *C. lavandulifolium*, Ecan *E. canadensis*, Hann *H. annuus*, Alap *Arctium lappa*, Cend *Cichorium endivia*, Cint *C. intybus*, Lsat *L. sativa*, Stac *Scaevola taccada*, Dcar *Daucus carota* subsp. *sativus*, Rchi *R. chinensis*, Mdom *Malus domestica*, Ntab *Nicotiana tabacum*, Slyc *S. lycopersicum*, Csat *Camelina sativa*, Atha *A. thaliana*, Taes *T. aestivum*, Osat *O. sativa*, Atri *A. trichopoda*, Gmon *G. montanum*, Ppat *Physcomitrium patens*, Acap *Adiantum capillus-veneris*, Crei *Chlamydomonas reinhardtii*. **B** Distribution of *LHC* members across the 24 plant species. Open circles indicate no genes are identified. **C** Frequency distribution of highly similar *LHC* gene pairs (≥ 55% identity) within specific identity ranges in eight species. **D** Distribution of Ks values for intraspecific *LHC* gene pairs (≥ 55% identity) in eight species. The vertical dashed line marks Ks = 0.5. The grouping legend on the right side of panel (**D**) also applies to panel (**C**)

Genomes of 24 representative plant species—spanning green algae (1), bryophyte (1), pteridophyte (1), gymnosperm (1), basal angiosperm (1), monocots (2) and dicots (17) were used to identify *LHC* superfamily members (Table S2, Fig. 1A). The superfamily size ranged from 23 to 158 members across these species. The *Lhc* subfamily was the largest, followed by *Lil* subfamily (*OHP*, *SEP*, *ELIP* and *Psb33*), whereas the *PsbS* and *FCⅡ* subfamilies retained highly conserved, low copy numbers (Fig. 1B, Table S6). Notably, the *LHC* superfamily exhibited a clear expansion within the examined Asteraceae lineages compared to core eudicots like *A. thaliana* (33), *Solanum lycopersicum* (43), and *Rosa chinensis* (35). Specifically, *A. annua* (54–65 members across different haplotypes) and its relatives, including *A. argyi* (95), *Chrysanthemum lavandulifolium* (82), *Lactuca sativa* (62), and *Helianthus annuus* (64), carried significantly more *LHC* members, largely due to expansions within the *Lhc* and *ELIP* subfamilies. While *Triticum aestivum* (158) possessed an exceptionally high number of genes due to its hexaploid nature, the counts in *O. sativa* (23), *Amborella trichopoda* (33), *Gnetum montanum* (23), and other species remained small to intermediate (Fig. 1B, Table S6).

To investigate the expansion of the *LHC* superfamily in Asteraceae, pairwise sequence similarity analysis was conducted among *LHC* members from four representative Asteraceae species and four non-Asteraceae core eudicot species. Although the overall similarity distributions were comparable across all species (Fig. S1), a clear divergence emerged when analyzing gene pairs with ≥ 55% sequence identity. The Asteraceae genomes showed a significantly greater abundance of these highly similar pairs (21.77% in *A. argyi* to 45.93% in *A. annua*) than non-Asteraceae plants (15.80% in *R. chinensis* to 25.25% in *S. lycopersicum*) (Fig. 1C, Table S7). Notably, the Ks distribution for these highly similar pairs (≥ 55% identity) displayed a peak near Ks = 0.5 specifically in the Asteraceae species (Fig. 1D), confirming active recent duplication events. Collectively, these results suggest that recent, lineage-specific duplications drove the expansion of the *LHC* superfamily in *A. annua* and its close relatives relative to other core eudicots.

### Tandem duplication-driven expansion and allelic diversity of the *LHC* superfamily in *A. annua*

**Fig. 2:**
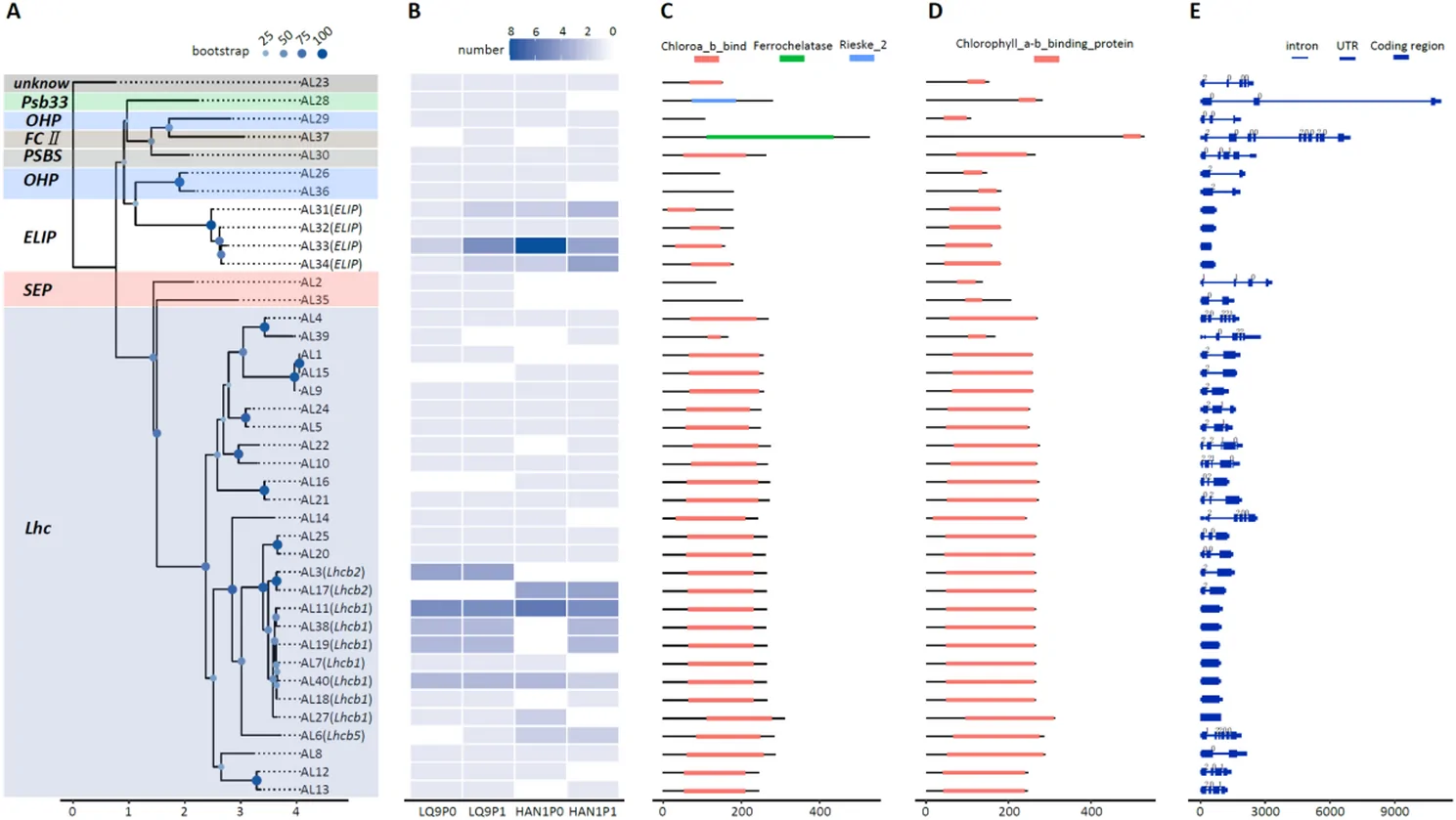
Phylogenetic relationship and structural features of *AaLHC* alleles across four haplotype genomes. **A** Maximum-likelihood (ML) phylogenetic tree of AaLHC proteins. Branches are colored by subfamily, and bootstrap support values are indicated at key nodes. **B** Copy number distribution of *AaLHC* alleles in the four haplotype genomes. **C** PFAM domains of AaLHC proteins. **D** SUPERFAMILY annotations of AaLHC proteins. **E** Gene structures of *AaLHC* alleles. Blue rectangles represent the coding sequences, thin blue lines connecting two exons represent introns, and flanking medium-thick bars represent 5′ or 3′ untranslated regions (UTRs)

A total of 229 *LHC* members were identified from four haplotype genomes of two *A. annua* strains [37]. Synteny analysis classified 205 of these members into 40 allelic groups (named AL1-AL40), leaving 24 as haplotype-specific loci (Fig. 2A, Table S8 and S9). Tandem duplication events were detected at 11 loci, encompassing 190 members. The number of *AaLHC* members varied substantially among the different haplotype genomes, driven by the presence/absence of specific allelic loci as well as variations in tandem copy numbers within individual allelic loci (Fig. 2B, Table S8). Notably, the *AaLhcb* (*AaLhcb1*, *AaLhcb2*, *AaLhcb5*) and *AaELIP* subfamilies exhibited prominent tandem expansions, with copy numbers per locus ranging from 2 to 8 (Fig. 2B, Table S8). These proteins ranged from 108 to 529 aa in length, with molecular weights between 10.01 and 59.21 kDa (Table S9). Most *AaLHC* members contained the characteristic Chloroa_b_bind domain (PFAM id PF00504), except for the *AaFCⅡ* and *AaPsb33* subfamilies (Fig. 2C). All identified members were annotated as Chlorophyll A-B binding proteins in the SUPERFAMILY database (Fig. 2D). Seven members were classified as pseudogenes due to disrupted gene structures or premature translation termination (Table S9).

Although gene structures varied from 1 to 10 exons across the superfamily, high structural conservation was observed within each *A. annua* subfamily (Fig. 2E). Strikingly, all *AaLhcb1* and *AaELIP* genes featured a uniform single-exon structure, distinct from the multi-exon configurations of other subfamilies (Fig. 2E, Table S9). Across broader eudicots, *Lhcb1* members displayed variable exon numbers. While *ELIP* homologs in the core eudicot *A. thaliana* and the Asteraceae-sister species *S. taccada* (Goodeniaceae) retained multi-exon structures, all analyzed Asteraceae *ELIPs* consistently possessed a single-exon architecture (Table S9 and Table S10). Given the immense size of the Asteraceae family and the limited taxa representation in current genomic datasets, this phylogenetic distribution tentatively suggests that the single-exon architecture might have arisen during or after the early diversification of Asteraceae. However, further genomic data from broader Asteraceae species, particularly those from basal lineages, are required to definitively resolve the ancestral state. Additionally, *AaELIP* copies shared high sequence similarity both within and between loci (Table S10). Phylogenetic analysis showed that *ELIPs* from the same plant family generally clustered into family-specific monophyletic clades (Fig. S2), reflecting independent evolutionary trajectories of *ELIP* genes across divergent plant families.

### Evolutionary origin and dynamic diversification of single-exon *AaELIPs*

**Fig. 3:**
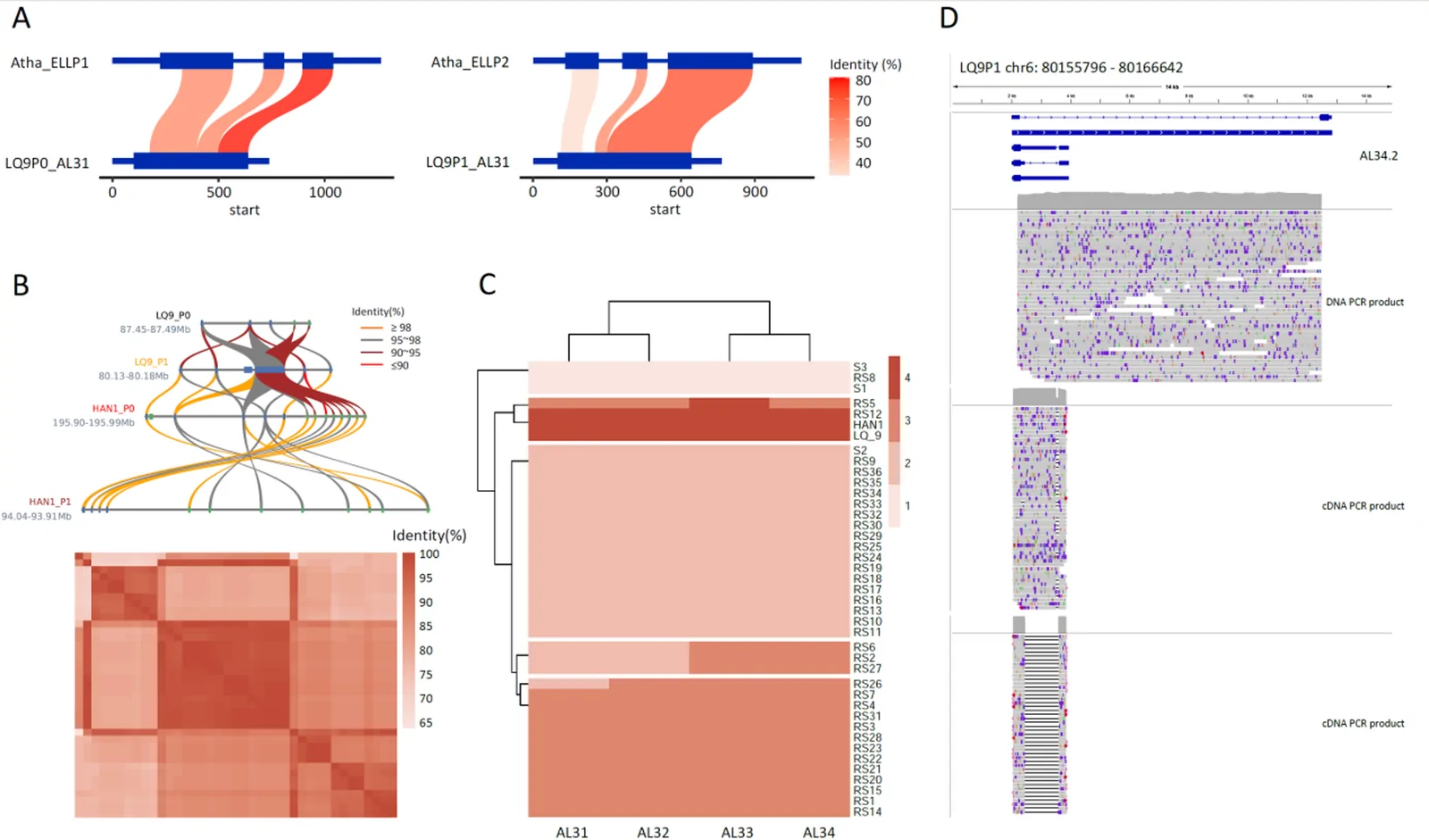
Comparative and population genomic analysis of *AaELIPs*. **A** Gene structure and sequence alignment of *ELIP* homologs between *A. annua* and *A. thaliana*. Blue rectangles represent the coding sequences (CDS), thin lines connecting two exons represent introns, and flanking medium-thick bars represent 5′ or 3′ UTRs. **B** Microsynteny and sequence similarity matrix of *ELIPs* across four *A. annua* haplotype genomes. The upper diagram illustrates the microsynteny among the *ELIP* loci using the LQ9P0 genome as a reference, with links connecting collinear gene pairs colored by sequence identity thresholds. The lower heatmap presents the pairwise protein sequence identity matrix among all identified *ELIP* proteins. **C** Heatmap of estimated copy number at core *ELIP* loci across 41 diverse *A. annua* individuals. **D** Genomic architecture and transcript profiles of a specific structurally disrupted *ELIP* allele (AL34.2) in LQ9P1 haplotype, characterized via Nanopore long-read sequencing. The alignment tracks display genomic DNA PCR mapping (confirming the physical insertion of an ultra-long intron at the genomic level) alongside reverse transcription PCR (RT-PCR) mapping (illustrating the structures of distinct long transcript isoforms generated at the transcript level)

To elucidate the evolutionary origin of the single-exon architecture of *AaELIPs*, a comparison of *ELIP* genes was performed between *A. annua* and *A. thaliana*. The single-exon region of *AaELIPs* showed high sequence similarity and direct spatial correspondence to the concatenated multi-exon regions of *A. thaliana* orthologs (Fig. 3A). Notably, no sequence homology to the *ELIP* introns of the sister lineage *S. taccada* (Goodeniaceae) was detected within the *A. annua* genome, and the flanking regions of *AaELIPs* lacked characteristic retrotransposition hallmarks, such as target-site duplications (TSDs) and poly(A) tails. Together, this direct coding-region homology, along with the lack of intron remnants and transposition signatures, strongly supports a lineage-specific intron elimination model rather than retrotransposition, although the exact underlying mechanism remains to be fully elucidated.

Despite the conserved single-exon architecture across Asteraceae, the *AaELIP* subfamily exhibited substantial evolutionary dynamism. Microsynteny analysis revealed that tandem duplication copy numbers varied from 5 to 13 across allelic loci (Fig. 3B). Pairwise sequence identity among all duplicated and allelic copies ranged from 69.78 to 100% both within individual loci and across different genomes (Fig. 3B, Table S11). Furthermore, population resequencing of 41 diverse *A. annua* individuals further uncovered extensive copy number variations, with 1 to 4 copies detected per locus (Fig. 3C, Table S12). Together, these polymorphic patterns highlight the active and ongoing evolution of the *ELIP* subfamily in *A. annua*.

This dynamism is further highlighted by a specific structural variant (AL34.2) within the LQ9P1 genome, where an ultra-long sequence insertion (~ 10 kb) disrupted the canonical single-exon structure (Fig. 3D). Genomic read mapping confirmed that this locus was not an assembly artifact. Although structurally disrupted, this locus remained transcriptionally active. Integrated analysis of RNA‑seq, full-length transcript, and PCR data demonstrated the production of three distinct long transcript isoforms, including a 1,949 bp intronless transcript, a 1,857 bp transcript with a 92 bp intron, and an 807 bp transcript harboring a 1,142 bp intron (Fig. 3D). All three isoforms lacked the characteristic LHC domain, whereas their closest sequence homolog retained an intact gene structure and produced normal mRNA. Given the current lack of functional validation, it remains to be elucidated whether these aberrant transcripts represent functional novel proteins, act as regulatory long non-coding RNAs (lncRNAs), or reflect transcriptional noise from a pseudogene undergoing evolutionary degradation. Collectively, these phenomena—intron elimination, dynamic CNVs, and ongoing structural disruptions—demonstrate that the *ELIP* subfamily in *A. annua* is undergoing remarkably active evolutionary transitions.

### *AaELIPs* responded specifically to UV-B stress with varied degrees among duplicated copies

**Fig. 4:**
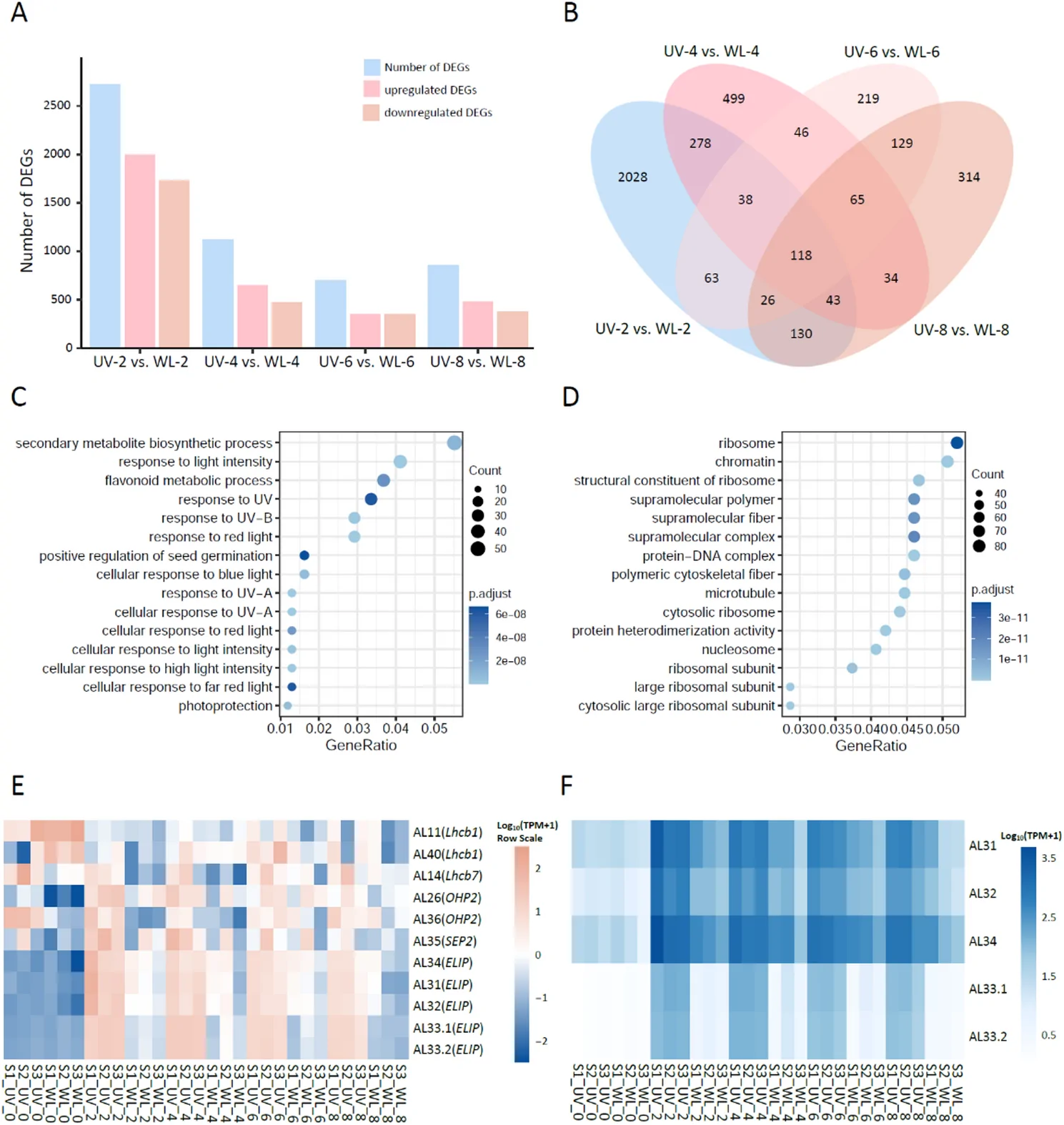
Transcriptomic response and functional enrichment of *A. annua* under UV-B stress. **A** Number of DEGs between UV-B and WL light treatments at four time points (2, 4, 6, and 8 h). **B** Venn diagram of overlapping DEGs across the four comparisons. **C**,** D** GO enrichment of (**C**) upregulated and (**D**) downregulated DEGs. **E** Heatmap of 11 core *LHC* superfamily DEGs based on row Z-score scaled Log_10_(TPM + 1). **F** Unstandardized heatmap illustrating the absolute expression levels for five individual *ELIP* loci

Although the photoprotection role of *ELIPs* under HL stress is well characterized, their specific functions in *A. annua* remain unclear [38–40]. Comprehensive profiling of *AaELIPs* expression across diverse conditions revealed strong induction by light, especially UV irradiation (Table S4, Fig. S3). To dissect this response, *A. annua* plants were subjected to UV-B stress for various durations, followed by transcriptomic sequencing. A 2 h exposure elicited the most pronounced global transcriptional shift, and a total of 1,648 upregulated and 2,396 downregulated DEGs were identified across all comparisons (Fig. 4A). Functional enrichment analysis showed that upregulated DEGs were primarily involved in light signaling (UV, blue light, red light, and HL), secondary metabolite and photoprotection (Fig. 4C), whereas downregulated DEGs were primarily associated with basal metabolic processes such as ribosome assembly and chromatin organization (Fig. 4D).

Among the 118 core DEGs shared across all UV-B time points,11 belonged to the *LHC* superfamily, including *Lhcb1*, *Lhcb7*, *OHP2*, *SEP2*, and multiple *ELIP* loci (Fig. 4B). Notably, although all *AaELIP* loci exhibited a highly synchronized temporal induction trend under UV-B stress (Fig. 4E), their absolute expression levels diverged (Fig. 4F and Fig. S4). Alleles from the same locus (*LQ9P0_AL33.1* and *LQ9P0_AL33.2*) clustered together with lower absolute expression, whereas alleles from *LQ9P0_AL31*, *LQ9P0_AL32* and *LQ9P0_AL34* formed a separate cluster characterized by markedly higher transcript abundance (Fig. 4F and Fig. S4). This expression divergence likely reflects a functional division into major and auxiliary roles within the expanded cluster.

While the response of *ELIPs* to light stress is evolutionarily conserved across plants (Table S4 and Fig. S5), the *AaELIP* subfamily has undergone a pronounced expansion defined by remarkable genomic and transcriptional dynamism—including extensive tandem duplications, structural streamlining into a single-exon architecture, and population-level copy number variations. Collectively, these findings may represent a key evolutionary adaptation that enhances plant capacity to cope with intense light and radiation stress in its native habitats.

## Discussion

This study systematically investigated the *LHC* superfamily across 24 plant species through comparative genomics and in-depth analysis of four haplotype genomes from two *A. annua* strains. Our findings revealed the lineage-specific expansion patterns, evolutionary dynamics, and UV-stress response mechanisms of the *LHC* superfamily, with a specific focus on the *ELIP* subfamily in *A. annua*. Specifically, an expansion of the *LHC* superfamily within *A. annua* and its close relatives was observed, which was predominantly driven by recent duplication events. In *A. annua*, the *ELIP* subfamily exhibited an extensive tandem duplication (Fig. 2B). Such lineage-specific, tandem duplication-driven expansions of *ELIPs* have been observed in various other stress-tolerant organisms, notably in resurrection plants like *T. ruralis* [10] and *Boea hygrometrica* [41, 42], as well as in certain evergreen gymnosperms such as pines and spruces [43], to counteract severe environmental constraints. Across extensive plant research, the functional conservation of *ELIPs* in mediating photoprotection is confirmed, as observed in the chlorophyte *C. reinhardtii* [12], the bryophyte *T. ruralis* [10], and the angiosperm *A. thaliana* [13]. Beyond their predominant responsiveness to light stress, *ELIP* subfamilies are also universally recognized for integrating signals from other diverse abiotic stress, showing marked induction under cold and drought conditions in species like *B. hygrometrica* [41, 42] and gymnosperms [43]. Consistently, in *A. annua*, the tandemly duplicated *AaELIP* loci were highly responsive to UV-B treatment, while simultaneously exhibiting prominent expression divergence among individual members, suggesting a potential role for *AaELIPs* in photoprotection.

In plants, a core mechanism of photoprotection under excessive irradiance involves NPQ and the efficient sequestration of free pigments [9]. Previous research has demonstrated that *ELIPs* specifically localize to PSII-enriched thylakoid domains near the vulnerable D1 protein, serving as a temporary reservoir to capture free chlorophylls released during light-induced protein degradation. By transiently coordinating these highly reactive molecules alongside complementary carotenoids, *ELIPs* dissipate excess excitation energy via static NPQ pathways, thereby suppressing the generation of hazardous ROS [9]. In *A. annua*, the prominent tandem expansion and expression divergence of *AaELIPs* likely function as an augmented molecular buffer network, thereby multiplying the capacity for rapid pigment sequestration and thermal dissipation to ensure robust stabilization of the photosynthetic machinery under severe radiation stress. However, the specific mechanisms still await experimental verification.

Notably, the *AaELIP* subfamily exhibited a highly distinctive genomic feature characterized by a uniform single-exon gene architecture (Table S9, Fig. 2E). Comparative sequence alignments against the model dicot *A. thaliana* and genomic analyses revealed that this streamlined single-exon architecture more likely arose from ancestral intron elimination rather than retrotransposition events, but the exact underlying mechanism remains to be further investigated. From an adaptive perspective, intron splicing is a complex and rate-limiting step in eukaryotic gene expression as maintaining and splicing introns imposes a substantial energetic cost and kinetic delay [44, 45]. Furthermore, under acute environmental stress, the cellular splicing machinery can be severely inhibited or prone to processing errors, leading to aberrant transcripts and limiting the expression of functional multi-exon genes [46, 47]. Based on the uniformly evolved single-exon structure of *AaELIPs* in *A. annua*, we speculate that this single-exon architecture allows for the rapid production of photoprotective proteins under stress. Bypassing the RNA splicing process significantly accelerates the translation of *AaELIP* transcripts, which allows *A. annua* to quickly produce large amounts of *ELIP* proteins to protect its photosynthetic machinery from rapidly changing light and UV-B radiation without time or energy delays. Interestingly, alongside these intact loci, a structurally disrupted *ELIP* allele retaining transcriptional activity was identified (Fig. 3D), however, due to the current lack of functional validation, it remains unclear whether these transcripts encode new functional proteins, act as regulatory lncRNAs, or simply reflect transcriptional noise from a degrading pseudogene.

Furthermore, only a few individuals exhibit minor variations in copy number. To eliminate potential confounding effects of sequencing depth on these observed variations, PCR-free libraries were utilized to minimize amplification bias. Mapping the resequencing reads from the 41 *A. annua* samples back to the reference genome revealed a consistently high and highly uniform genome coverage ranging from 85.64% to 88.39% across all sequenced individuals. This exceptional alignment homogeneity demonstrates that the genomic datasets possess strong inter-sample comparability, thereby ensuring that the minor fluctuations reflect genuine, low-frequency population polymorphisms rather than sequencing anomalies. Besides, the expression variations observed among different *AaELIP* members under UV-B treatment suggest a potential functional specialization or gene dosage effects. However, these patterns represent differences only at the transcriptional level and do not directly demonstrate actual differences in biological function. Given the lack of direct experimental evidence, the specific functional roles of individual *AaELIP* loci remain unclear, which is a major limitation of our current work. From a broader perspective, decoding the genomic architecture of medicinal plants provides critical insights into the genetic basis of traditional Chinese medicine (TCM) quality [37, 48, 49]. In TCM species, gene copy number variations and tandem duplications frequently serve as a genomic reservoir that buffers environmental stress, which directly shapes the accumulation and stability of secondary metabolites [50, 51]. Our findings on the tandemly expanded *AaELIP* cluster highlight how genomic structural variations can potentially partition stress-responsive roles, offering a valuable reference for understanding environmental adaptation and stress resilience in wild *A. annua* populations. Therefore, future transgenic functional experiments are planned to fully clarify the specific roles of these *AaELIP* genes in stress tolerance.

## Conclusion

In conclusion, this study provides a comprehensive genome-wide analysis of the *LHC* superfamily across 24 plant species and highlights the unique evolutionary dynamics of the *ELIP* subfamily in *A. annua*. Our findings demonstrate that recent tandem duplication events drove a significant expansion of *AaELIPs*, resulting in allelic diversity and dynamic copy number variations among populations. Structurally, the uniform single-exon architecture of *AaELIPs* likely arose from ancestral intron elimination, while the presence of a transcriptionally active, disrupted allele highlights that this subfamily is undergoing active evolutionary transitions. Under UV-B stress, *AaELIP* loci show a highly synchronized temporal induction trend but clear expression divergence, suggesting a functional specialization or gene dosage effects. This dramatic tandem expansion and structural streamlining are hypothesized to represent a key evolutionary adaptation, which may enhance the capacity of *A. annua* to cope with intense light and radiation stress. Overall, these insights provide a valuable foundation for future research to fully decode the molecular mechanisms underlying *A. annua*’s adaptation to its complex light environments.

## Supplementary Information

Supplementary Material 1: Fig. S1. Density distribution of sequence similarity among *LHC* genes within species. Fig. S2. Neighbor-Joining phylogenetic tree of *ELIP* genes from 24 plant species. Fig. S3. Heatmap of *AaELIP* expression under various treatments. Fig. S4. (A) Pearson correlation analysis of five *ELIP* genes expressions. Color gradient indicates correlation coefficients. Significance markers based on two-tailed tests: *P < 0.05, **P < 0.01, ***P < 0.001. (B) Protein sequence similarity matrix of five *ELIP* genes. (C) Expression dynamics of five *ELIP* genes under UV-B treatment. (D) Expression dynamics of five *ELIP* genes under WL treatment. Fig. S5. Heatmap of *A. thaliana ELIP* expression under various treatments.

Supplementary Material 2: Table S1. Multi-database identification and classification criteria of the LHCs in *Arabidopsis thaliana*. Table S2. Species used in phylogenetic analysis. Table S3. Resequencing samples of *A. annua*. Table S4. Primers used for LQ9P1_AL34.2 structural variations. Table S5. Transcriptome data used in this study. Table S6. The number of *LHC* superfamily genes in 24 plant species. Table S7. Number and proportion of highly similar *LHC* gene pairs (≥55% identity) in eight plant genomes. Table S8. *LHC* alleles in four haplotypes. Table S9. Characteristics of *LHC* members. Table S10. Distribution of exon numbers of *ELIPs* in other eudicot. Table S11. Similarity matrix of *ELIP* allelic among the four haplotypes. Table S12. Copy number evaluation in *A. annua* population.

## Data Availability

The LHC gene sequences were deposited in the Global Pharmacopoeia Genome Database [19] at http://www.gpgenome.com/species/92. Data supporting the findings of this work are available within the paper and its Supplementary information files.

## Abbreviations

LHC: Light-harvesting chlorophyll a/b-binding
ELIP: Early light-induced protein
NPQ: Non-photochemical quenching
ROS: Reactive oxygen species
OHPs: One-helix proteins
SEPs: Stress enhanced proteins
Lils: LHC-like proteins
PSII: Photosystem II
CNVs: Copy number variations
HL: High-light
CDD: Conserved Domain Database
pI: Isoelectric point
MW: Molecular weight
ONT: Oxford Nanopore Technologies
IGV: Integrative Genomics Viewer
RH: Relative humidity
WL: White light
UV-B: Ultraviolet-B
DEGs: Differentially expressed genes
GO: Gene Ontology
ML: Maximum-likelihood
TSDs: Target-site duplications
lncRNAs: Long non-coding RNAs
CDS: Coding sequences
UTRs: Untranslated regions
RT-PCR: Reverse transcription PCR
