# Supplementary material for: Tandem duplication-driven expansion and UV-B stress adaptation of the LHC gene family in Artemisia annua L
Source: BMC Plant Biol. 2026 Jun 29;26:1588. doi: 10.1186/s12870-026-09370-4 (PMC13576340; doi:10.1186/s12870-026-09370-4)
Supplement: Supplementary file 1 [file 12870_2026_9370_MOESM1_ESM.docx]

**Supplementary Figures**

Fig. S1. Density distribution of sequence similarity among *LHC* genes within species.

Fig. S2. Neighbor-Joining phylogenetic tree of *ELIP* genes from 24 plant species.

Fig. S3. Heatmap of *AaELIP* expression under various treatments.

Fig. S4. (**A**) Pearson correlation analysis of five *ELIP* genes expressions. Color gradient indicates correlation coefficients. Significance markers based on two-tailed tests: *P < 0.05, **P < 0.01, ***P < 0.001. (**B**) Protein sequence similarity matrix of five *ELIP* genes. (**C**) Expression dynamics of five *ELIP* genes under UV-B treatment. (**D**) Expression dynamics of five *ELIP* genes under WL treatment.

Fig. S5. Heatmap of *A. thaliana ELIP* expression under various treatments.

**Fig. S1**. Density distribution of sequence similarity among *LHC* genes within species.

**Fig. S2** Neighbor-Joining phylogenetic tree of *ELIP* genes from 24 plant species.

**Fig. S3** Heatmap of *AaELIP* expression under various treatments.

**Fig. S4** (**A**) Pearson correlation analysis of five *ELIP* genes expressions. Color gradient indicates correlation coefficients. Significance markers based on two-tailed tests: *P < 0.05, **P < 0.01, ***P < 0.001. (**B**) Protein sequence similarity matrix of five *ELIP* genes. (**C**) Expression dynamics of five *ELIP* genes under UV-B treatment. (**D**) Expression dynamics of five *ELIP* genes under WL treatment.

**Fig. S5** Heatmap of *A. thaliana ELIP* expression under various treatments.
